# Anatomical and histological classification of the stellate ganglion: implications for clinical nerve blocks

**DOI:** 10.1007/s00276-024-03533-4

**Published:** 2024-12-11

**Authors:** Rarinthorn Samrid, Mona King, Jacie Pujol, Miguel Angel Reina, Joe Iwanaga, R. Shane Tubbs

**Affiliations:** 1 Department of Anatomy, Faculty of Medicine, Khon Kaen University, Khon Kaen, Thailand; 2 Department of Neurosurgery, Tulane Center for Clinical Neurosciences, Tulane University School of Medicine, 131 S. Robertson St. Suite 1300, New Orleans, LA 70112 USA; 3 Department of Neurology, Tulane Center for Clinical Neurosciences, Tulane University School of Medicine, New Orleans, LA USA; 4 Department of Structural and Cellular Biology, Tulane University School of Medicine, New Orleans, LA USA; 5 Tulane University School of Medicine, New Orleans, LA USA; 6 Department of Neurosurgery and Ochsner Neuroscience Institute, Ochsner Health System, New Orleans, LA USA; 7 Dental and Oral Medical Center, Kurume University School of Medicine, 67 Asahi-machi, Kurume, Fukuoka Japan; 8 Division of Gross and Clinical Anatomy, Department of Anatomy, Kurume University School of Medicine, 67 Asahi-machi, Kurume, Fukuoka Japan; 9 Department of Anatomical Sciences, St. George’s University, St. George’s, Grenada; 10 Department of Surgery, Tulane University School of Medicine, New Orleans, LA USA; 11 University of Queensland, Brisbane, Australia; 12 Department of Anesthesiology, CEU-San Pablo University School of Medicine, Madrid-Montepríncipe University Hospital, Madrid, Spain; 13 Department of Anesthesiology, University of Florida College of Medicine, Gainesville, FL USA

**Keywords:** Cervicothoracic ganglion, Anatomy, Histology, Sympathetic, Nerve blockade, Horner’s syndrome

## Abstract

**Purpose:**

The stellate ganglion (SG), or cervicothoracic ganglion, is usually located anterior to the neck of the first rib. Various techniques, such as ultrasonographic imaging and fluoroscopic approaches, are used to assist in the anesthetic blockade of the SG. However, there are reported complications associated with SG block; some patients had medication-related or systemic side effects, and some had procedure-related or local side effects. So, understanding the anatomy of the SG is critical for diagnosis and treatment of nerve block accuracy and to avoid unnecessary nerve damage during surgical procedures. This study aimed to collect data for the gross shape of the SG and histologically investigate these different types.

**Methods:**

The SG from 31 formalin-fixed adult cadavers (59 sides) were studied. The prevalence and shape of the SG were recorded and photographed. Next, the SG for each type was examined histologically.

**Results:**

The SG were classified into four types based on their shape: dumbbell, spindle, star, and inverted L shapes. The frequency of each type was as follows: spindle (47.46%), dumbbell (27.12%), star (23.73%), and L-inverted shapes (1.69%). Each type had a similar number of nerve cell bodies. Interestingly, the inverted-L shaped SG was histologically, discontinuous but grossly fused.

**Conclusion:**

An improved understanding of the SG’s macro and microanatomy can help better understand patient presentations and improve clinical and surgical results in procedures performed near this important neck structure.

## Introduction

The stellate (cervicothoracic) ganglion (SG) is located along the sympathetic trunk anterior to the neck of the first rib. It travels along the anterior surface of the longus colli muscle behind the subclavian artery. At its inferior end, the ganglion is related to the cervical pleura and vertebral artery [23]. Arising from the T1-L2 segments of the spinal cord, the sympathetic fibers enter the sympathetic trunk through the white rami communicantes. Preganglionic sympathetic fibers ascend superiorly in the sympathetic trunk and may synapse in one of three cervical ganglia (superior, middle, or inferior) or the upper thoracic ganglia. After synapsing in a sympathetic ganglion, the postganglionic fibers synapse on their target tissue [19].

In approximately 80% of the population, the SG is formed from the fusion of the inferior cervical and first thoracic sympathetic ganglia and sometimes the second thoracic ganglia [3, 8]. The SG carries preganglionic sympathetic fibers ascending to the cervical sympathetic trunk, as well as postganglionic sympathetic fibers, which supply sympathetic activity to the upper limbs, cardiac tissues, and head in conjunction with the cervical sympathetic trunk [23]. The shape of the ganglion has been reported as dumbbell shaped, spindle shaped, and an inverted-L shape [17, 22].

Stellate ganglion blockade (SGB) is commonly used to treat complex localized pain syndromes of the head, neck, and upper limbs [11]. Also, SGB can treat patients with palmar hyperhidrosis [9]. The landmark for localizing the SGC is the anterior tubercle of the transverse process of the C6 vertebra (or Chassaignac’s tubercle) [10]. This level is used as the C7 level is very near the cervical pleura, thus increasing the risk of pneumothorax [11]. Other complications include vessel injury and hoarseness [6, 28].

Various adjuncts have been used to help block the SG, such as ultrasonographic imaging [14, 21, 30] and fluoroscopy [1]. Ultrasound-guided SGBs have a reported complication rate of around 25% [5, 10, 24, 25]. Therefore, our cadaveric study aimed to improve our understanding of the SG’s macro and microanatomy to prevent complications.

## Materials and methods

The SG was investigated in 31 formalin-fixed cadavers (59 sides, 15 males and 16 females). The mean age at death was 78.5 years (52–98 years). Three sides, one male and two female, were not included in this study as the area of the SG was damaged due to previous dissection. In the supine position, the SG was located at the anterior border of the first rib or C7 vertebral level posterior to the subclavian artery and posterolateral to the prevertebral fascia along the anterior surface of the longus colli muscle. The shape of the SG was recorded and photographed. The SG was then classified based on its shape. Lastly, samples from each shape were randomly harvested and fixed in 10% formalin, paraffin-embedded, sectioned with a thickness of 5 μm, and stained using hematoxylin and eosin (H&E) visualize under a light microscope (EVOS FL auto-imaging system, ThermoFisher Scientific, Waltham, Massachusetts, USA).

The authors hereby confirm that every effort was made to comply with all local and international ethical guidelines and laws concerning the use of human cadaveric donors in anatomical research [13].

## Results

### Classification of the stellate ganglia

**Fig. 1:**
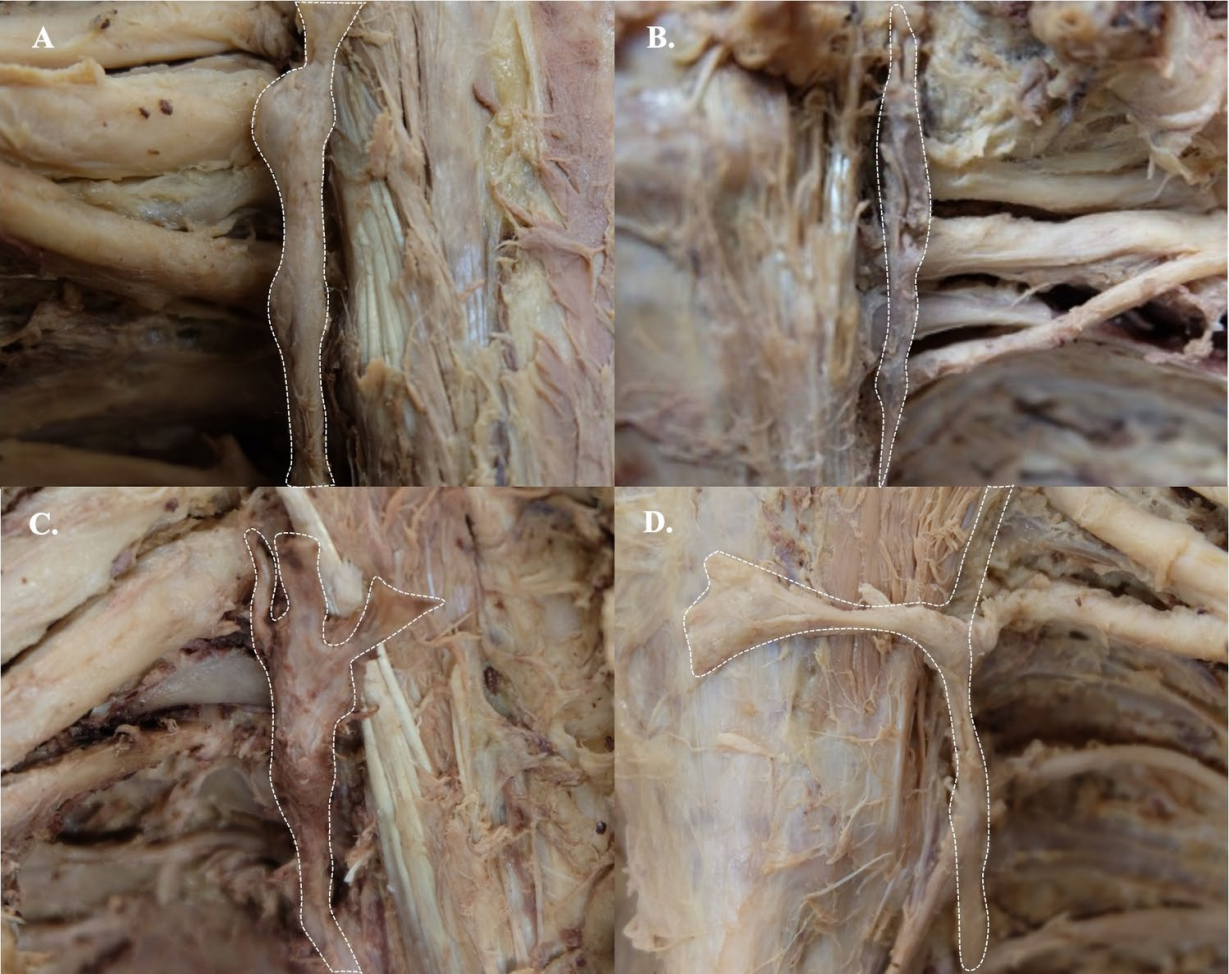
Shapes of the stellate ganglia **A** dumbbell-shaped, **B** spindle-shaped, **C** star-shaped, and **D** inverted L-shaped

Of the 59 SG, 25 were located at the neck at the C7 transverse process and 34 at the neck of the first rib. These ganglia were a fusion between the inferior cervical and the first thoracic sympathetic ganglion. No contributions from the second thoracic sympathetic ganglion were identified. The SG was classified into four types, i.e., dumbbell, spindle, star, and inverted L-shapes (Fig. 1). The most common type was the spindle-shape (47.46%), followed by the dumbbell (27.12%), star (23.73%), and L-inverted shapes (1.69%). Out of 29 sides from males, 13 SG were spindle-shaped (44.83%), 10 were star-shaped (34.48%), and six were dumbbell-shaped (20.69%). No inverted L-shaped ganglia were found in male specimens. Of the 30 female sides, 15 SG were spindle-shaped (50%), 10 were dumbbell-shaped (33.33%), four were star-shaped (13.33%), and 1 was an inverted L-shape (3.33%) (Table 1).

**Table 1:** Summary of stellate ganglions type

Type	Total (*n* = 59)	Male (*n* = 29)	Female (*n* = 30)
Dumbbell shape	16 (27.12%)	6 (20.69%)	10 (33.33%)
Spindle shape	28 (47.46%)	13 (44.83%)	15 (50%)
Star shape	14 (23.73%)	10 (34.48%)	4 (13.33%)
Inverted-L shape	1 (1.69%)	None	1(3.33%)

## Histological examination of the stellate ganglia

**Fig. 2:**
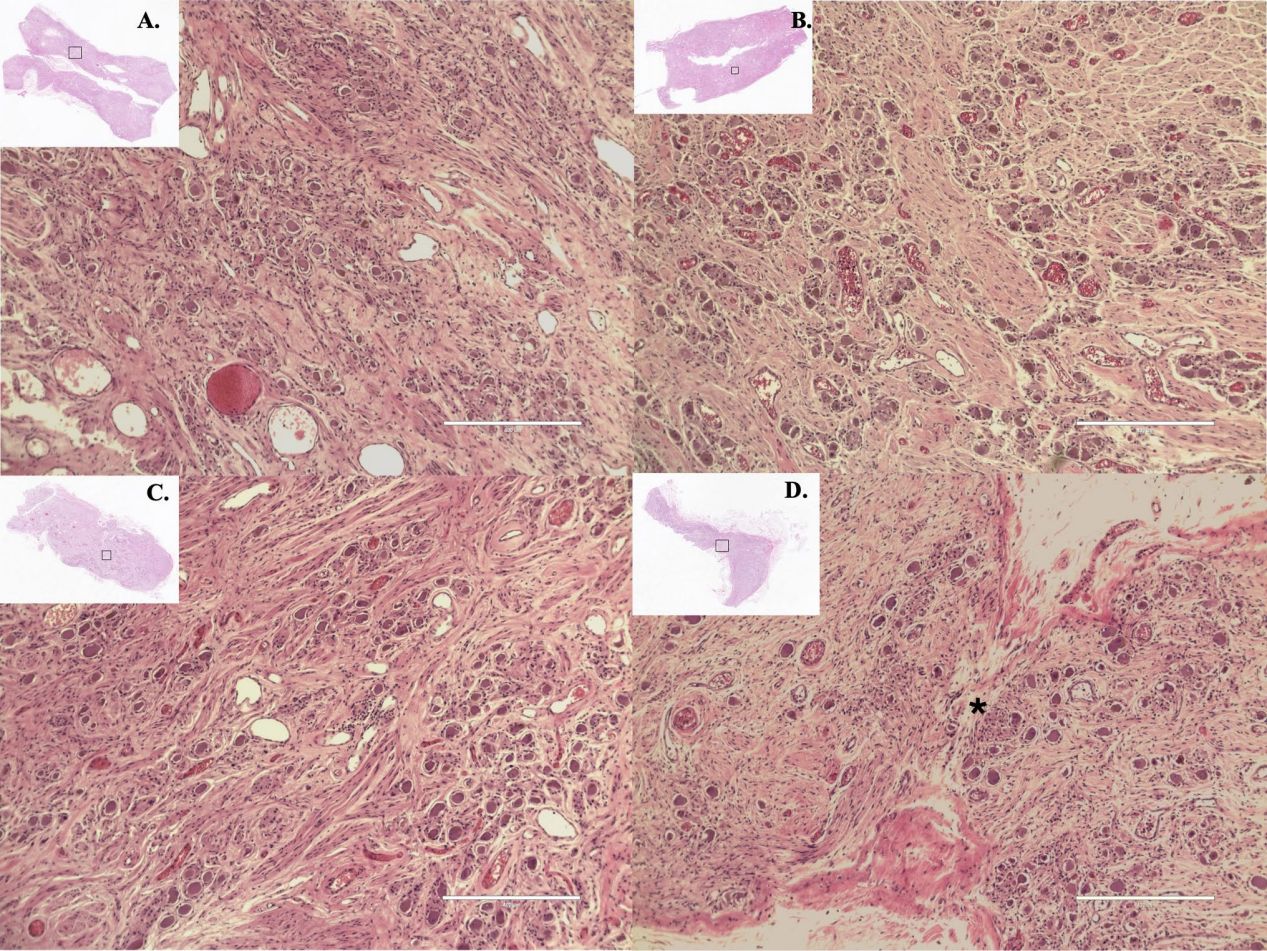
Histological characteristics of stellate ganglia stained with hematoxylin and eosin (40x). **A** dumbbell-shaped, **B** spindle-shaped, **C** star-shaped, and **D** inverted-L shape. The connection point between the ganglia (*). Scale bar: 400 μm

Histological analysis was performed to determine the characteristics of all SG subtypes. A similar number of nerve cell bodies were found in the SG of all types (Fig. 2). Connective tissue and blood vessels surrounded the SG’s nerve cell bodies and satellite cells. In one female specimen with an inverted L-shape ganglion, histologically, the ganglion was discontinuous and thus did not represent a true fusion of the inferior cervical and first thoracic ganglia, although grossly, this was its appearance (Fig. 2D).

## Discussion

Various shapes of the SG have been reported (Table 2.) [2, 15, 20, 22]. For example, Pather et al. found a spindle-shaped ganglion on 21, a dumbbell shaped ganglion on 20 sides, and an inverted-L shaped ganglion on 34 sides [22]. However, the present study found four different shapes of the SG: dumbbell-shaped on 16 sides, spindle-shaped on 28 sides, star-shaped on 14 sides, and one side, an inverted L-shape. The star-shaped ganglia, which is the namesake of the SG, made up the minority of shapes for this structure.

**Table 2:** Prevelence of shape of stellate ganglion

Authors	Years	Type of stellate ganglion	Prevalence
Panther et al.	2006	Spindle Dumbbell Inverted-L	28% 26.7% 45.3%
Dabuzinskiene et al.	2011	Spindle Dumbbell Inverted-L	56% 24% 20%
Kwon et al.	2018	Fusiform elongated Fusiform rounded Bilobed	51.7% 13.8% 34.5%
Mhetre et al.	2024	Fusiform elongated Fusiform rounded Bilobed	67.5% 3.75% 28.75%

Various techniques for performing SGB have been used. Historically, a blind technique using palpation of superficial landmarks was used [16, 26]. The success of SGB is based on the presence of ipsilateral Horner’s syndrome and an increase in the temperature of the affected limb [18, 29]. However, ultrasound-guided SGB at C7 still risks vertebral artery injury and pneumothorax [5, 6, 27]. The anesthetic injected toward the SG can spread to other areas via loose connective tissues [3, 4]. As the various shapes of the SG have been implicated as contributing to unsuccessful SGB [14], our study is important to increasing our knowledge of the gross and microscopic anatomical variations of the SG.

The vagus and recurrent laryngeal nerves travel near the SG; thus, if inadvertently blocked or stimulated, hoarseness and/or cough could result [6]. Although there are a variety of techniques used for SGB, such as the ultrasonographic imaging technique [14, 21, 24] and fluoroscopy [1], complications still occur. Using ultrasound guidance for SGB, one study found diffuse staining from SGB spread along the prevertebral space from C4 – T1 levels [7]. In the present study, the histology of an inverted L-shaped SG highlights that grossly, the prevalence of the SG might be overestimated as, histologically, there was discontinuity of the inferior cervical and first thoracic ganglia with this type, but grossly, it appeared as a single fused ganglion.

## Conclusion

This study presented the macro and microanatomy of the SG. In one female specimen with an inverted L-shape ganglion, histologically, the ganglion did not represent a true fusion of the inferior cervical and first thoracic ganglia. This finding improve our anatomical understanding of this structure, which might help clinicians prevent complications during invasive procedures.

### Limitations

Prevalence of the SG was calculated based on the gross anatomical dissection, not histology. So true prevalence of fusion might be overestimated. The age of cadavers might affect the results as the age of the cadavers used in this study was elderly (mean:78.5 years, range: 52–98 years).

## Data Availability

No datasets were generated or analysed during the current study.
